# RNA‐Based Therapies for Inherited Metabolic Disorders

**DOI:** 10.1002/jimd.70150

**Published:** 2026-02-01

**Authors:** Reddy Sreekanth Vootukuri, Sonam Gurung, Roopkatha Ghosh, Philippa B. Mills, Julien Baruteau, Haiyan Zhou

**Affiliations:** ^1^ Genetics and Genomic Medicine University College London Great Ormond Street Institute of Child Health London UK; ^2^ NIHR Great Ormond Street Hospital Biomedical Research Centre London UK; ^3^ Great Ormond Street Hospital for Children NHS Foundation Trust London UK

**Keywords:** AON, gene silencing, inborn errors of metabolism, mRNA, n‐of‐1, RNA therapy, siRNA

## Abstract

Inherited metabolic disorders (IMDs) are a diverse and complex group of genetic conditions resulting from deficiencies in enzymes, transporters, or cofactors. These deficiencies lead to metabolic dysfunction and severe clinical consequences. Despite significant progress in understanding their molecular basis, treatment options remain limited for many IMDs. RNA‐based therapies including antisense oligonucleotides (AONs), small interfering RNAs (siRNAs), and messenger RNA (mRNA) therapeutics have emerged as promising treatment strategies for modulating gene expression, silencing pathogenic transcripts, and restoring deficient proteins, offering new avenues for disease intervention. In this review, we summarise the chemistry and mechanisms of action of different RNA therapy modalities including splice‐modulating and gene silencing AONs, siRNAs, and mRNA therapies. The delivery of these RNA‐based therapies remains a significant challenge. Here, we outline the development of various delivery methods, including lipid nanoparticle (LNP) packaging, ligand conjugation, and tissue‐specific delivery systems as well as their clinical applications in treating IMDs. We also summarise the clinical application of RNA therapies in rare diseases, an area that has grown rapidly in the last few years, as exemplified by the success of some n‐of‐1 therapies for IMDs, which have redefined personalised medicine by enabling rapid, patient‐specific drug development. As RNA‐based therapeutics continue to evolve, their clinical applications in IMDs will require continued innovation in novel chemistries, advanced delivery technologies, and streamlined regulatory frameworks to unlock their full potential.

## 1 Introduction

Inherited metabolic disorders (IMDs) represent a diverse group of genetic conditions characterised by defects in enzymes, transport proteins or cofactors. As of June 2024, 1564 IMDs have been recognised [1], and this number is expected to grow substantially, potentially at an accelerating pace, as advances in genomic technologies and data interpretation continue to uncover novel gene‐disease associations. IMDs disrupt metabolic pathways or organelle functions, resulting in improper processing of biochemical substances [2, 3]. They often manifest in infancy or childhood, presenting with symptoms ranging from developmental delay to severe organ failure, which can be life‐threatening without timely intervention [4, 5]. Although each IMD is individually rare, collectively they affect approximately 1 in 800 to 1 in 1000 live births, underscoring their clinical and public health significance [6, 7]. IMDs can arise from enzyme deficiencies that lead to the accumulation of toxic substrates or deficient products, as seen in organic acidurias, vitamin B_6_ metabolism disorders, urea cycle disorders, lysosomal storage disorders, and glycogen storage diseases [8, 9, 10, 11, 12]. Additionally, chronic‐transporter deficiencies, which result in metabolite accumulation in organelles, are exemplified by conditions such as X‐linked adrenoleukodystrophy (XALD) (OMIM #300100) and familial hypercholesterolemia (FH) (OMIM #143890) [13, 14]. Together, these disorders illustrate the complex and multifaceted nature of IMDs, which pose significant diagnostic and therapeutic challenges.

While this large group of monogenic disorders is amenable to disease‐modifying treatments, a recent study reported that only 18% of all currently known IMDs are treatable [15]. Traditional management strategies for IMDs rely heavily on dietary modifications, enzyme replacement therapies (ERT) and small‐molecule drugs [16, 17, 18]. While these approaches have provided life‐saving benefits, they often involve lifelong commitments to treatment and diet restriction, immune‐related complications, and challenges associated with organ transplantation, emphasising the need for more transformative treatments [19, 20, 21]. For many IMDs, there are no approved disease‐modifying treatments.

RNA‐based therapies introduce a promising paradigm shift in treating IMDs by addressing the genetic cause of these disorders. These therapies leverage advanced techniques such as antisense oligonucleotides (AONs), small interfering RNAs (siRNAs), and messenger RNA (mRNA) therapies. AONs can degrade mRNA or modulate pre‐mRNA splicing to influence protein expression; siRNAs silence specific genes through the RNA interference (RNAi) pathway, and mRNA therapies provide transient but effective protein replacement. Driven by advancements in RNA chemistry, targeted delivery technology, and a deeper understanding of molecular biology, these modalities offer unprecedented precision, minimal invasiveness, and potential curative solutions for IMDs.

Recent clinical applications illustrate the transformative potential of RNA therapies in managing metabolic disorders and highlight their broad therapeutic potential. For instance, inclisiran, an siRNA‐based therapy, has demonstrated efficacy in reducing low density lipoprotein (LDL) cholesterol in patients with hypercholesterolemia (FH) (OMIM #143890) by silencing the expression of proprotein convertase subtilisin/kexin type 9 (*PCSK9*) mRNA [22, 23]. Similarly, mipomersen, an AON targeting apolipoprotein *B‐100* mRNA, significantly lowered LDL cholesterol in individuals with homozygous familial hypercholesterolemia [24], showcasing the promise of RNA‐based interventions. Moreover, mRNA‐3927, encoding human propionyl‐CoA carboxylase (PCC), represents a ground‐breaking mRNA therapy for the treatment of propionic acidemia (OMIM #606054), where interim results from clinical trials have shown data in favour of clinical benefit, although further evidence is needed to confirm these findings [25, 26]. Many RNA therapies face challenges, however, including long‐term safety concerns, tissue specific delivery, and immune response to foreign elements. Mipomersen was withdrawn from the market after just a few years following concerns about hepatotoxicity risks.

Advances in molecular biology, biotechnological innovations and novel RNA‐based therapies are however expanding the horizons for treating IMDs, introducing innovative strategies that complement and extend the capabilities of traditional siRNAs, AONs and mRNA therapies. This review will discuss the mechanisms of action and clinical applications of AONs, siRNAs and mRNA therapies, highlighting ongoing research aimed at enhancing the potential of these technologies and optimising delivery methods. It will also summarise n‐of‐1 therapies poised to revolutionise IMD treatment.

## 2 AON and siRNA Therapy for IMDs

### 2.1 Mechanism of Action of AONs

The term ‘antisense’, referring to the non‐coding strand complementary to the protein‐coding sense strand, was first introduced by Zamecnik and Stephenson in 1978 when they developed a 13‐nucleotide long oligonucleotide inhibitor targeting Rous sarcoma virus [27]. AONs are short, single‐stranded DNA or RNA molecules, typically 13–25 nucleotides long, with most designs ranging from 18 to 22 bases, to ensure specific binding to target RNA sequences through complementary base‐pairing [28, 29, 30]. Their mechanism of action is determined by their design and chemical modifications, which enhance stability, binding affinity, specificity, and cellular uptake. They can modulate gene expression in various ways, including inducing RNA degradation via endogenous Ribonuclease H (RNase H) and non‐RNase H mediated mechanisms.

#### 2.1.1 RNA Degradation via Endogenous RNase H

AONs are commonly used to exploit the RNA‐cleaving activity of RNase H, a conserved family of non‐sequence specific enzymes responsible for recognising and cleaving RNA–DNA hybrids such as R‐loops [31, 32]. AONs for this purpose are engineered in a gapmer configuration, with a central region of phosphorothioate DNA nucleotides (the ‘gap’) flanked by chemically modified RNA‐based nucleotides at each end (the ‘wings’), typically in a 5–10–5 pattern. The DNA gap is essential for forming a hybrid with target pre‐ or mRNA and activating RNase H1, a subtype of RNases H. RNase H1, equipped with its unique hybrid‐binding domain, specifically recognises the DNA–RNA hybrid and cleaves the RNA strand at the site of hybridisation [33, 34]. This initial cleavage, resulting in unprotected termini, sets off a cascade of RNA degradation mediated by cellular exoribonucleases. These include XRN1, which processes RNA from the 5′ to 3′ end, and the exosome complex, comprising key components such as EXOSC10 and DIS3, which degrade RNA through the 3′ to 5′ decay pathway, ensuring thorough depletion of the target RNA [35, 36]. This mechanism bypasses the need for translational machinery, offering a precise approach for reducing target RNA levels.

To optimise these AONs for therapeutic use, specific chemical modifications are employed to enhance their stability, interaction with RNase H1 and resistance to degradation. One of the most critical modifications is the introduction of phosphorothioate (PS) linkages, where a non‐bridging oxygen in the phosphodiester backbone is replaced with sulphur. This substitution significantly increases resistance to nuclease degradation and enhances enzymatic stability, thereby improving interactions with RNase H1 [37]. PS‐modification improves AON pharmacokinetics by facilitating the binding of the AON to plasma proteins and improving its circulation time in the bloodstream. This may lead to low renal clearance [38] and prolonged persistence in vivo, which are associated with enhanced efficacy but also potential toxicity [39]. PS linkages can also reduce on‐target binding specificity, which may contribute to off‐target effects and unintended interactions, further influencing toxicity profiles [40]. Despite these challenges, PS modifications remain a cornerstone of oligonucleotide therapeutics, providing a foundation upon which additional chemical enhancements can be built to improve safety.

Other AON modifications that enhance nuclease resistance and improve their pharmacokinetic properties include modifications at the 2′ position of the ribose sugar in the flanking regions of the AON. This includes replacement of the 2′‐OH in ribose with an O‐methyl group or an O‐methoxyethyl group. These modified AONs are referred to as 2′‐O‐methyl (2′‐OMe) and 2′‐O‐methoxyethyl (2′‐MOE) modified AONs, respectively [41, 42, 43]. 2′‐OMe modification prevents degradation of the AON by endogenous RNases by sterically hindering access to the phosphodiester backbone; additionally, this modification reduces non‐specific effects by improving AON binding affinity and specificity, thereby minimising unintended interactions with off‐target RNA sequences [44]. 2′‐MOE modification enhances oligonucleotide stability and resistance by locking the ribose sugar in the C3′‐endo conformation, which strengthens hybridisation affinity and specificity for complementary RNA targets [45]. When combined with PS modification, these modified oligonucleotides (either in 2′‐MOE or 2′‐OMe) are resistant to endogenous RNase degradation [46].

Examples of AONs that utilise the RNase H mechanism to degrade target mRNA in the treatment of metabolic disorders include mipomersen and inotersen, both FDA‐approved drugs. Mipomersen targets ApoB‐100 to treat homozygous familial hypercholesterolemia, while inotersen silences transthyretin (TTR) to treat hereditary transthyretin amyloidosis (hATTR; OMIM #176300) [47].

#### 2.1.2 Non‐RNase H Mediated Mechanisms

##### 2.1.2.1 Blocking Translational Elements

Translational arrest by AONs can be achieved by targeting key elements in mRNA that influence ribosomal access and efficiency. Translation initiation begins with ribosome recruitment to the 5′ cap, followed by ribosome scanning towards a start codon to initiate protein synthesis. This process is often influenced by secondary structures or regulatory elements within the 5′ untranslated region (UTR), such as iron responsive elements (IREs), G‐quadruplexes, and internal ribosome entry sites, which can range from a few to thousands of base pairs [48, 49, 50, 51]. AONs can bind to these translationally relevant regions, acting as steric barriers to ribosomal access and inhibiting translation, or they can disrupt inhibitory structures to enhance translation. Similarly, AONs can target upstream open reading frames (uORFs), complex secondary structures, or binding sites for regulatory proteins to modulate translation efficiency. By masking uORFs or altering RNA folding, AONs can modulate ribosomal entry, promoting or inhibiting protein synthesis as needed [52, 53, 54]. Genetic variants within uORFs may be capable of contributing to phenotypes as described in loss of function pathogenic variant carriers. For example, a stop‐strengthening uORF variant in *SHMT2* has been nominally associated with cardiac and movement disorders [55]. Such findings underscore the role of uORFs in gene regulation and their potential as therapeutic targets for AONs.

##### 2.1.2.2 Splicing Modulation

AONs can modulate splicing by targeting specific sequences on pre‐mRNA, such as splice sites or splicing regulatory elements to induce exon inclusion or skipping. This approach allows precise control of alternative splicing while bypassing RNA degradation pathways. AONs achieve this by binding to exon‐intron boundary regions, including the conserved 5′ splice site, where nucleophilic attack from the branch point initiates lariat formation and the 3′ splice site, where exon ligation occurs [56, 57]. Additionally, AONs can also target the branch point, an adenine‐containing splicing signal upstream of the 3′ splice site, or the polypyrimidine tract, which recruits spliceosomal components [58]. By masking or altering regulatory elements like exonic or intronic splicing enhancers (ESEs and ISEs) and silencers (ESSs and ISSs), AONs can induce either exon‐skipping or exon‐inclusion [59, 60, 61]. In the future, high‐throughput sequencing, computational modelling, and experimental validation will help identify precise exonic and intronic elements that regulate splicing, offering deeper insights into these processes. These elements may serve as potential targets for AONs, enabling the production of specific desirable mRNA isoforms tailored to therapeutic needs.

For non‐RNase H mediated mechanisms, RNA 2′‐OH modifications such as 2′‐OMe or 2′‐MOE or stabilising the ribose with locked nucleic acid (LNA) modifications throughout the AON sequence evade RNase H1 activation, ensuring AONs do not degrade the target RNA but instead modulate splicing. These chemical modifications not only shield the 2′‐OH groups from nuclease activity but also improve pharmacokinetics and reduce potential toxicity and off‐target effects, enhancing the safety profile of AONs. Alternative backbones such as peptide nucleic acids (PNAs) and morpholinos offer high resistance to nucleases due to their uncharged nature, enhancing their longevity [62, 63].

Clinically, splice‐modulating AONs have shown success in treating genetic disorders. Eteplirsen, golodirsen, viltolarsen, and casimersen are phosphorodiamidate morpholino oligomers (PMO) that promote exon skipping in Duchenne muscular dystrophy (DMD) and restore the dystrophin reading frame [64, 65]. Nusinersen, in phosphorothioate 2′‐MOE chemistry, enhances *SMN2* exon 7 inclusion for spinal muscular atrophy (SMA) treatment [66]. Beyond DMD and SMA, splice‐modulating AONs are being developed for several of the IMDs, including phenylketonuria (PKU) (OMIM #261600) [67], 6‐pyruvoyl‐tetrahydropterin synthase deficiency (PTPSD) (OMIM #261640) [68], hereditary myopathy with lactic acidosis (OMIM #255125) [69], glycogen storage disease type 1a (GSD1a) (OMIM #232200) [70] and neuronal ceroid lipofuscinoses type 7 (CLN7), a form of Batten disease (OMIM #610951) [71].

**TABLE 1:** FDA approved and ongoing clinical trials of RNA therapies for inherited metabolic diseases.

Drug name	Target disease	Mechanism of action	Phase and ClinicalTrials.gov ID	Company
Patisiran	Hereditary transthyretin‐mediated amyloidosis (hATTR)	siRNA	Approved for marketing	Alnylam Pharmaceuticals
Inclisiran	Heterozygous familial hypercholesterolemia (HeFH)	siRNA	Approved for marketing	Novartis Pharmaceuticals
Givosiran	Acute hepatic porphyria (AHP)	siRNA	Approved for marketing	Alnylam Pharmaceuticals
Lumasiran	Primary hyperoxaluria type 1 (PH1)	siRNA	Approved for marketing	Alnylam Pharmaceuticals
Vutrisiran	Polyneuropathy of hereditary transthyretin‐mediated (hATTR) amyloidosis	siRNA	Approved for marketing	Alnylam Pharmaceuticals
Nedosiran (DCR‐PHXC)	Primary hyperoxaluria type 1 (PH1)	siRNA	Approved for marketing	Dicerna Pharmaceuticals Inc., a Novo Nordisk company
ABX1100	Late‐onset Pompe disease (LOPD)	siRNA	Early phase 1 NCT06109948	Aro Biotherapeutics
Nucresiran (ALN‐TTRSC04)	Transthyretin‐mediated amyloidosis (ATTR)	siRNA	Phase 1 NCT05661916	Alnylam Pharmaceuticals
Inclisiran (German Inclisiran network)	Hypercholesterinaemia	siRNA	Observational NCT05438069	Jena University Hospital
Inclisiran (Japanese participants)	Heterozygous familial hypercholesterolemia (HeFH)	siRNA	Phase 2 completed NCT04666298	Novartis Pharmaceuticals
DCR‐PHXC	Primary hyperoxaluria type 1 and type 2 (PH1 and PH2)	siRNA	Phase 2 completed NCT03847909	Novo Nordisk A/S
Inotersen	Hereditary transthyretin‐mediated amyloidosis (hATTR)	AON	Approved for marketing	Ionis Pharmaceuticals
Volanesorsen	Familial chylomicronemia syndrome (FCS)	AON	Approved for marketing	Ionis Pharmaceuticals
Eplontersen	Hereditary transthyretin‐mediated amyloidosis (hATTR)	AON	Approved for marketing	Ionis pharmaceuticals and Astrazeneca
ION283	Lafora Disease	AON	Phase1/2 NCT06609889	Berge Minassian
Eplontersen	Transthyretin (ATTR) amyloidosis	AON	Observational NCT06465810	AstraZeneca
CMP‐CPS‐001	Carbamoyl‐phosphate synthetase 1 (CPS1) deficiency	AON	Phase 1 NCT06247670	CAMP4 Therapeutics Corporation
Vupanorsen	Dyslipidaemia	AON	Phase2 completed NCT04516291	Pfizer
mRNA‐3927	Propionic acidaemia (PA)	mRNA	Phase I/II NCT04159103	Moderna
mRNA‐3705	Methylmalonic acidaemia (MMA)	mRNA	Phase I/II NCT04899310	Moderna
ARCT‐810	Ornithine transcarbamylase deficiency (OTCD)	mRNA	Phase II NCT06488313	Arcturus
mRNA‐3745	Glycogen storage disease 1a (GSD1a)	mRNA	Phase I/II NCT05095727	Moderna
mRNA‐320	Phenylketonuria (PKU)	mRNA	Phase I/II NCT06147856	Moderna (Withdrawn as of Sept 2025)
UX053	Glycogen storage disease type III (GSD III)	mRNA	Phase I/II NCT04990388	Ultragenyx Pharmaceutical Inc. (Terminated—not related to safety concerns)
mRNA 3351	Crigler‐Najjar Disease	mRNA	Preclinical	Moderna

### 2.2 Small Interfering RNAs (siRNAs)

siRNAs have emerged as powerful tools for gene silencing, leveraging the endogenous RNAi pathway, a natural defence mechanism conserved in plants, animals and some fungi. In 1998, the groundbreaking discovery by Fire, Mello and colleagues that double‐stranded small interfering RNA could silence gene expression in *Caenorhabditis elegans* established RNAi as a transformative biological mechanism [72]. This pathway protects against viruses, silences transposons and regulates gene expression [73, 74]. siRNAs are double‐stranded RNA molecules, typically processed from longer precursors into short 21–26 nucleotide siRNAs by enzymes such as Dicer and protein activator of PKR (PACT) [75]. These siRNAs are then incorporated into the RNA‐induced silencing complex (RISC), which processes them into active single strands to specifically target complementary mRNA for degradation [76, 77, 78]. This mechanism enables precision treatments for various diseases, including IMDs.

Therapeutic siRNAs are typically designed with a 19‐nucleotide duplex region and 2‐nucleotide overhangs (e.g., dTdT) on the 3′ end of each strand, a design that enhances stability and protects against exonuclease degradation [79, 80]. Delivery into cells is primarily achieved via endocytosis using methods such as lipid nanoparticles or ligand‐mediated uptake, with endosomal escape being a critical step to ensure siRNAs reach the intracellular RNAi machinery [81]. Alternative delivery methods, such as electroporation, bypass endocytosis and allow direct entry [82].

Upon activation of RISC, the passenger or sense strand of the siRNA duplex is removed, leaving the siRNA guide or antisense strand to direct RISC to target mRNA for post‐transcriptional gene silencing [83]. For siRNAs with perfect complementarity to the target mRNA, argonaute2 (AGO2), part of the RISC complex, catalyses cleavage of the mRNA. This cleavage exposes unprotected RNA ends, which are subsequently processed by the RNA surveillance machinery including XNR1 (a 5′ to 3′ exoribonuclease) and the 3′ to 5′ exosome complex. However, with partial complementarity, siRNAs induce translational repression rather than mRNA cleavage [84]. This can occur through two main pathways: one involves de‐adenylation by the carbon catabolite repression 4‐negative on TATA‐less (CCR4:NOT) deadenylase complex or the enzyme poly (A)‐specific ribonuclease (PARN), both of which shorten the poly (A) tail. This is followed by de‐capping mediated by the DCP1:DCP2 de‐capping complex and subsequent degradation by exonucleases [85, 86, 87, 88, 89]. The other pathway involves translational repression without mRNA degradation, where siRNAs inhibit ribosome assembly or sequester target mRNA into processing bodies (P‐bodies) for temporary storage or decay [90, 91].

The RISC complex is highly efficient, once the mRNA is cleaved RISC is recycled to target additional mRNAs and sustain silencing over time [92]. Notably, while chemical modifications are not strictly necessary for siRNA function in cell‐based work due to their mimicry of the endogenous RNAi pathway, modifications like PS linkages at siRNA termini can enhance stability in some contexts, although fully PS‐modified siRNAs are less active than their phosphodiester (PO) counterparts [93].

While AONs are more commonly associated with splicing modulation, siRNAs can also regulate splicing in certain scenarios. For instance, duplex RNAs targeting aberrant splice sites within exons or introns have been shown to alter splicing outcomes in genes such as *Dystrophin* or *SMN2* in an AGO2‐dependent manner [94, 95].

Clinically, siRNAs have demonstrated tremendous potential. Patisiran, the first FDA‐approved RNAi therapeutic (2018) (Table 1), targets *TTR* mRNA in the liver to reduce both mutant and wild‐type TTR protein production in hATTR amyloidosis. Similarly, givorisan, approved for acute hepatic porphyria (AHP) (OMIM #612740), silences *ALAS1* mRNA, preventing the accumulation of neurotoxic intermediates such as delta‐aminolevulinic acid (ALA) and porphobilinogen (PBG) (Table 1). Beyond these, emerging siRNA therapeutics are being developed for IMDs. These include the inhibition of glycogen synthesis in glycogen storage diseases through silencing hepatic *Gys2* [96], targeting *Gys1* and *CD71* (transferrin receptor type 1, TfR1) to restore glycogen balance in Pompe disease [97], inhibition of globotriaosylceramide (Gb3) synthase to reduce glycosphingolipid accumulation in Fabry disease [98] and inhibition of glucosylceramide synthase (GCS) as a potential therapeutic strategy for Gaucher disease [99], underscoring the transformative potential of siRNAs in addressing complex metabolic conditions.

Beyond therapeutic applications, AONs and siRNAs are invaluable tools for studying disease molecular mechanisms of IMDs. By selectively silencing genes or modulating splicing, these technologies enable researchers to dissect metabolic pathways and predict downstream effects of genetic changes. This approach provides critical insights into the pathophysiology of IMDs, allowing for the identification of novel therapeutic targets and a deeper understanding of pathway dynamics.

### 2.3 Delivery of siRNAs and AONs

The ease of delivery of siRNAs and AONs in vivo depends on the target organ. It can be hindered by multiple biological barriers including rapid clearance of native or unmodified AONs and siRNAs from the blood circulation, their susceptibility to nuclease degradation, as well as rapid elimination via renal excretion, rendering them unstable and unsuitable for therapeutic applications [100, 101] and challenges in achieving tissue‐specific targeting.

Advancements in chemical modifications to the RNA moiety to improve pharmacokinetics and evade immune defences, nanoparticle delivery systems and tissue targeting conjugates are helping to overcome these barriers. PS backbone modification and modifications to the 2‐OH groups such as 2′‐OMe and 2′‐MOE have been extensively employed to enhance nuclease resistance, improve stability and optimise pharmacokinetic properties. In addition, other modifications, including LNA, glycol nucleic acids (GNA), 2′‐fluro (2′‐F) and 5′‐vinylphosphonate, which are extensively reviewed in the literature, provide enhanced structural integrity [102, 103]. For example, 5′‐vinylphosphonate modifications to the siRNA at the 5′ end of the guide strand have been introduced to improve nuclease resistance and silencing efficacy [104]. The strategic placement and number of chemical modifications on AONs and siRNAs can significantly enhance their pharmacokinetic properties, including stability, biodistribution and cellular uptake.

To further improve therapeutic efficacy, a variety of delivery strategies have been developed to overcome biological barriers. Lipid nanoparticles (LNPs) have been investigated extensively not only for AON and siRNA delivery, but also for mRNA delivery and are discussed in more detail below. They have demonstrated significant potential in pre‐clinical and clinical applications, such as in delivering patisiran [105, 106, 107] and givosiran [108]. Another effective delivery strategy involves conjugating chemically modified AONs or siRNAs with N‐acetyl galactosamine (GalNAc), enabling targeted uptake into hepatocytes via the asialoglycoprotein receptor (ASGPR) [109, 110]. This receptor is highly expressed in the basolateral surface of the hepatocyte membrane and is primarily responsible for recognising and eliminating circulating glycoproteins with exposed galactose or GalNAc residues [111]. The binding of GalNAc to ASGPR triggers receptor‐mediated endocytosis, facilitating efficient and hepatocyte‐specific delivery. Among various versions of GalNAc ligands, the tri‐antennary GalNAc structure, conjugated to either the 5′ or 3′ end of modified oligonucleotides, has been identified as the most chemically refined and resistant to metabolic degradation for therapeutic use. This hepatocyte‐targeted delivery not only enhances efficacy but also reduces off‐target effects [112, 113, 114]. This strategy has been successfully applied in approved siRNA therapies such as inclisiran in hypercholesterolaemia or mixed dyslipidaemia and vutisiran in hATTR amyloidosis [115]. GalNAc conjugation has also markedly enhanced the efficacy of AON therapies. A notable example is eplontersen, a GalNAc‐conjugated version of inotersen used for the treatment of hATTR amyloidosis. This modification has optimised the dosing regimen, reducing the required dose from 300 mg weekly for inotersen to just 45 mg monthly for eplontersen while also significantly minimising adverse effects [116]. The advent of GalNAc conjugation has revolutionised the development of AON and siRNA therapies, particularly for IMDs where the liver is the primary target organ.

While liver‐targeting of siRNAs and AONs can be successfully achieved, delivery to extra‐hepatic targets remains an area of active research. For instance, siRNAs encapsulated in beta‐1,3‐D‐glucan particles (GeRPs) have demonstrated effective oral delivery to macrophages, successfully suppressing systemic inflammation by silencing Map4k in vivo [117]. Also, the development of the FORCE platform, which uses an antigen‐binding fragment highly specific to the human TfR1, enables the delivery of oligonucleotides to muscle [118]. Furthermore, the development of selective organ targeting (SORT) LNPs has shown potential in directing RNA therapeutics to specific tissues beyond the liver [119]. Similarly, strategies for brain delivery, such as transporter‐utilising nanocarriers, peptide conjugation or ligand‐targeted delivery systems, aim to overcome the blood–brain barrier obstacle for oligonucleotide delivery to the central nervous system [120, 121, 122, 123].

### 2.4 AON N‐OF‐1 Therapies in IMDs

Despite significant advances in genetic therapies, only 5% of rare diseases have FDA‐approved treatments, highlighting a significant gap in medical care due to unique regulatory challenges [124]. N‐of‐1 therapies are transforming the treatment landscape for ultra‐rare disorders by providing personalised solutions tailored to individual patients. These therapies precisely target genetic variants that may be unique to a single individual or a very small group of people. Positioned at the intersection between medical research and clinical care, N‐of‐1 strategies represent a novel approach to developing individualised treatments, where each therapy can be likened to a randomised controlled trial (RCT) conducted in a single patient [125, 126].

In this review, n‐of‐1 therapies refer to individualised treatments designed for a single patient. This usage is distinct from the formal n‐of‐1 trial design but can be considered within the broader framework of single‐case experimental designs (SCEDs), which also include n‐of‐few approaches.

AONs offer a highly targeted, customisable, and rapidly deployable therapeutic strategy that aligns well with the needs of n‐of‐1 treatments for ultra‐rare diseases, where personalised medicine is often the only viable option. This approach is well suited for severely debilitating or life‐threatening diseases where no alternative treatment options are available, and the conditions will be rapidly progressing, resulting in early death and/or devastating or irreversible morbidity within a short time frame without treatment. Compared to traditional small‐molecule drugs or biologics, AONs can be designed and synthesised relatively quickly. This is particularly important for ultra‐rare diseases, where time is often critical, and the patient population is too small to justify lengthy development timelines. Individualised RNA therapy may also serve as a research approach, where insights from individual cases contribute to broader medical knowledge and inform group‐level outcomes for other related conditions.

A pioneering example is milasen, an AON therapy developed for a child with the IMD, CLN7 Batten disease. Milasen, a 22‐nucleotide AON with PS and 2′‐MOE modifications, worked by correcting a splicing defect in *MFSD8*. Remarkably, this treatment was conceptualised, rigorously tested, and administered within 1 year, improving the patient's quality of life and setting a precedent for similar initiatives globally [71]. For n‐of‐1 or SCED approaches in IMDs, suitability depends on disease‐specific features. Key factors include the rate of clinical progression and disease severity, the availability of robust and quantifiable biomarkers, amenability of the underlying genetic defect with different RNA‐based therapeutic approaches, and the opportunity to intervene before irreversible pathology develops [127, 128]. Together, these criteria provide a framework for identifying IMDs that are most amenable to individualised RNA therapy development.

Following the promise of milasen, further systemic research to promote n‐of‐1 AON therapy has been conducted, including development of relevant FDA guidelines [129], a potential framework for individualised splice‐switching AON for ataxia‐telangiectasia (AT) (OMIM #208900) [130], guidelines for eligibility assessment of pathogenic variants suitable for AON treatment [127], and the development of a robust patient‐derived iPSC modelling system for scalable AON screening [131].

The status of AON n‐of‐1 therapy in IMDs is still at an early stage. Ongoing advancements in AON technology, delivery, genetic diagnosis and regulatory support are paving the way for broader adoption and rapid clinical translation in this field.

## 3 mRNA Therapy for IMDs

The development and commercial success of mRNA vaccines Comirnaty (Pfizer‐BioNTech) and Spikevax (Moderna) for preventing SARS‐CoV‐2 infection during the COVID‐19 pandemic marked a significant milestone for mRNA‐based therapeutics [132, 133]. mRNA technology has evolved significantly since its initial discovery to the development of in vitro‐transcribed (IVT) mRNA and the first proof‐of‐concept study in animals in 1990. The technology over time has since expanded beyond prophylactic vaccines (e.g., against infectious diseases) to include therapeutic vaccines (e.g., against cancer) and protein replacement therapies. These advances offer promising solutions for cancer, cardiovascular diseases, regenerative medicine, and rare genetic diseases including IMDs [134, 135, 136]. Interestingly, the pipeline of mRNA therapies in development is focused primarily on targeting rare diseases. mRNA therapy is one of the most common RNA‐based therapeutic modalities used currently [137].

### 3.1 mRNA Technology and the Delivery System

**FIGURE 1:**
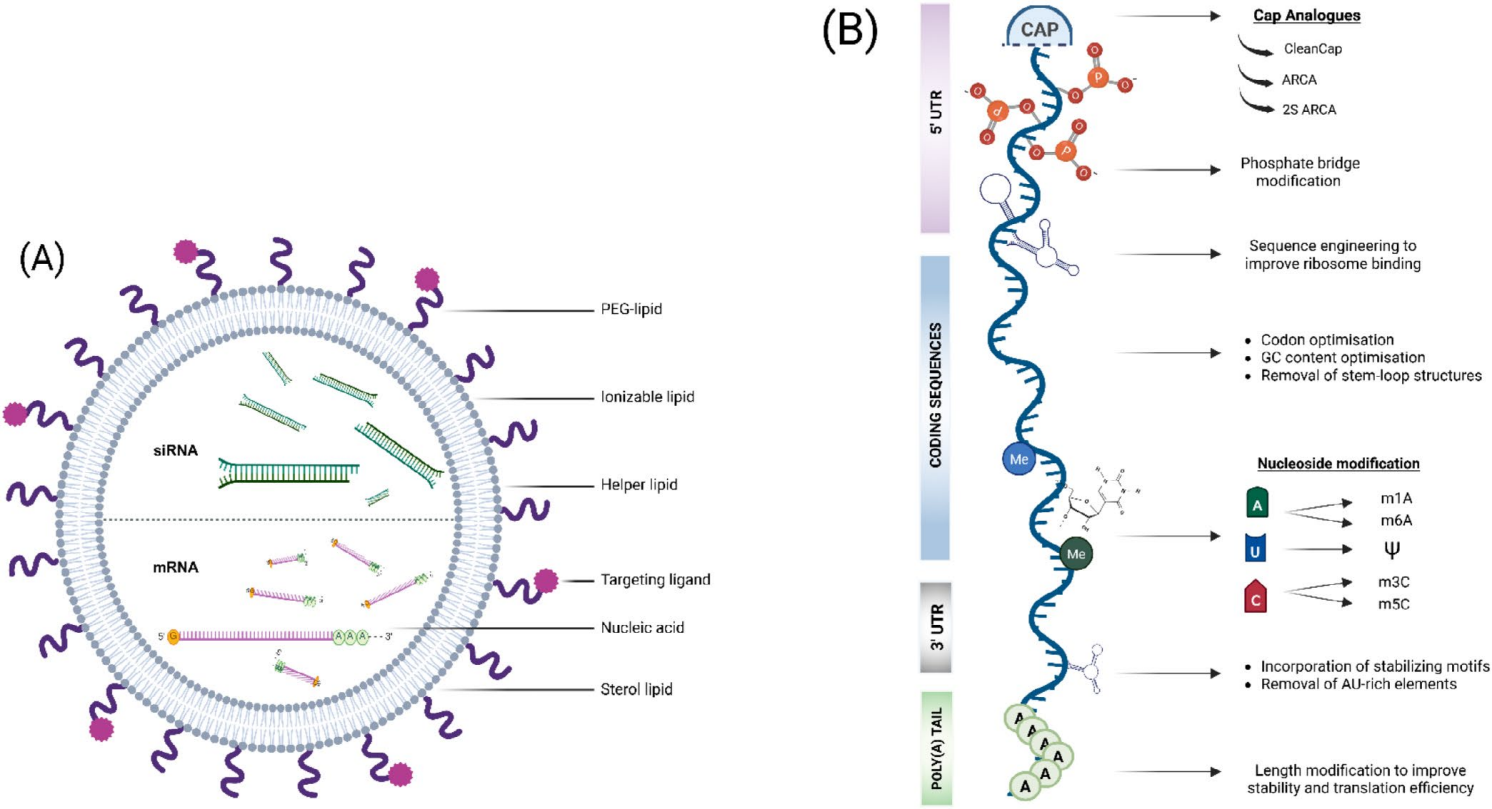
Lipid nanoparticles (LNP) and mRNA optimisation. (A) mRNA and siRNA cargoes are encapsulated in lipid nanoparticles, which are comprised of ionisable lipids, helper lipids, PEGylated lipids and cholesterol, alongside targeting moieties. (B) Multiple mRNA optimisation strategies include modifications to key structural elements: 5′ cap, 5′ and 3′ untranslated regions (UTRs), open reading frame (ORF) and polyadenylated (poly(A)) tail for enhanced translatability, reduced immunogenicity and improved stability.

Breakthroughs in mRNA engineering [138] and synthesis through in vitro transcription (IVT) have significantly enhanced translatability, reduced immunogenicity, and improved stability [139]. The modified mRNA product, when delivered into target cells, is translated into the desired protein by the cellular machinery [140, 141]. This allows for post‐translational modifications and subcellular localisation to occur while eliminating the need for costly protein manufacturing [140]. Furthermore, this strategy bypasses some key limitations of traditional gene therapy, such as the requirement for nuclear entry, synthesis of a complementary DNA strand for single‐stranded DNA viral vectors, and the risks associated with immunogenicity and genomic integration [136]. The technologies underlying mRNA therapy and its delivery vehicle to target cells, mainly by lipid nanoparticles (LNPs) (Figure 1A) for IMDs, have been extensively reviewed in a recent article in this journal [142]. Therefore, we will only briefly summarise the key points here and refer the reader to this review for further details.

mRNA engineering for therapeutic use can optimise various key structural elements: the 5′ cap, 5′ and 3′ UTRs, open reading frame (ORF) and polyadenylated (poly(A)) tail (Figure 1B). These optimisations, alone or in combination, can improve mRNA stability, extend its half‐life and increase protein expression by enhancing ribosomal engagement. The incorporation of biochemically modified nucleosides such as N1‐ and N6‐methyladenosine (m1A, m6A), 3‐methylcytosine (m3C), 5‐methylcytosine (m5C), pseudouridine (Ψ) and 2′‐O‐methylation (Nm) in mRNA enhances stability and reduces innate immunogenicity [143]. Use of synthetic cap analogues provides higher affinity for translation initiation factors like eukaryotic translation initiation factor 4E (eIF4E), thus enhancing protein expression and resistance to exonucleases [144, 145]. Sequence engineering of the 5′‐and 3′‐UTRs also enhances mRNA half‐life and translation [146], while the design of the mRNA coding sequence focuses on codon optimisation, increasing GC content and incorporating peptides to protect mRNA from degradation and enhance functionality [136]. Modifications to the poly(A) tail, including changes in its length and chemical substitutions like guanylation, help prevent deadenylation and further enhance mRNA stability [147]. While these optimisation strategies have been instrumental in advancing the clinical translation of mRNA therapeutics, the development of effective delivery systems is crucial to ensuring mRNA reaches its target cells and maintains its therapeutic efficacy in vivo.

**FIGURE 2:**
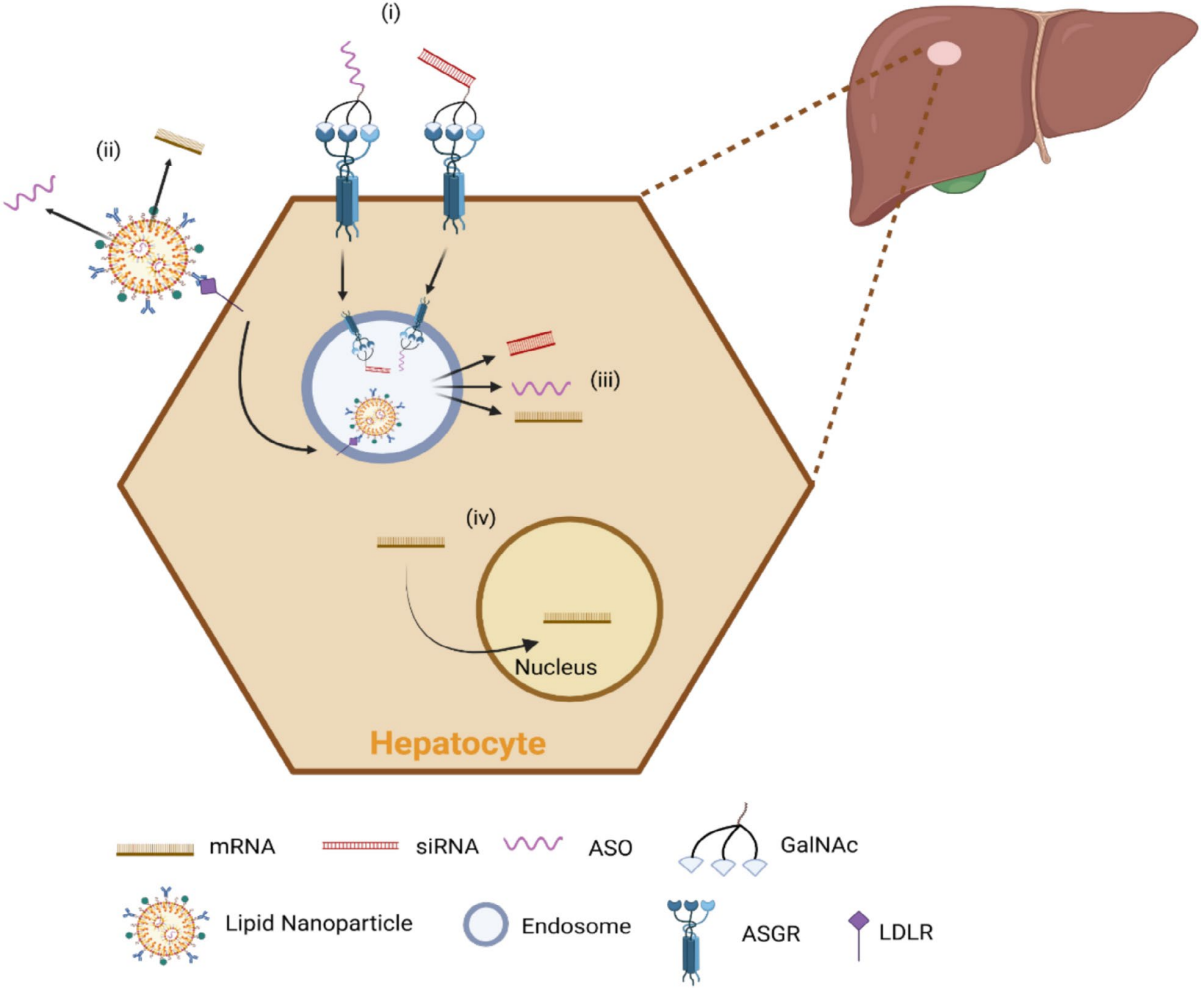
Hepatocyte‐targeted RNA therapies. (i) GalNAc‐mediated hepatocyte‐targeted delivery of siRNA or ASOs. (ii) ApoE‐mediated uptake of LNPs into hepatocytes. mRNA and siRNA can be encapsulated in hepatocyte‐targeted LNPs. (iii) mRNA, siRNA, or ASOs are released into the cytosol likely via endocytosis and endosomal escape. (iv) Cytosol‐released ASOs can freely diffuse into the nucleus. ASGR, asialoglycoprotein; ASO, antisense oligonucleotide; GalNAc, N‐acetylgalactosamine; LDLR, low‐density lipoprotein receptor; siRNA, small interfering RNA.

RNA therapies, including not only mRNA but also siRNAs and AONs, have been delivered using lipids, lipoplexes and polymers of various compositions, with LNPs emerging as an effective platform (Figure 2). This is exemplified by the siRNA‐based therapeutic patisiran for hATTR and the mRNA‐based COVID‐19 vaccines [148, 149]. A typical LNP consists of four key lipid components: ionisable or cationic lipids that encapsulate and protect the mRNA cargo, polyethylene glycol (PEG)‐lipids that enhance stability and prolong circulation, structural phospholipids that support functions such as endosomal escape, and cholesterol for added stability (Figure 1). Liver tropism of LNP is high due to apolipoprotein E (ApoE) binding the surface of LNPs, which facilitates uptake by LDL receptors [150, 151, 152]. Interestingly, non‐LDL receptor‐mediated uptake of LNPs by hepatocytes has also been demonstrated through use of GalNAc‐conjugated LNPs, which targeted the hepatocytes through GalNAc interaction with the ASGPR, a targeting strategy successfully implemented for AONs [153]. Different strategies have been developed to modify the liver‐biased biodistribution and/or limit off‐target effects. This is essentially achieved by modifying lipid chemistry or composition in LNPs or incorporating protein ligands or antibodies for tissue‐specific targeting [154]. Overall, LNPs offer advantages including simple formulation, biocompatibility, high cargo capacity and favourable pharmacokinetics [155], with advancements in engineering and microfluidics improving their safety, efficacy and scalability [148, 156].

### 3.2 Preclinical Progress

mRNA therapy, compared to alternative enzyme replacement strategies, has shown a significantly higher efficacy, with LNPs being the preferred vehicle for delivery. LNPs are easily taken up by hepatocytes, making liver IMDs an ideal therapeutic target for this technology. As such, preclinical studies employing a mRNA‐mediated protein replacement strategy using the mRNA‐LNP system in the landscape of liver IMDs have shown great promise [141]. Due to the short mRNA half‐life and the transient nature of mRNA therapy, kinetics following a single injection are assessed to determine the interval between each repeated dose. On average, re‐administration in preclinical models is performed every 3–7 days, with doses ranging from 0.5 to 2 mg/kg [157, 158, 159, 160, 161, 162, 163]. The intravenous route of administration is preferred for efficient liver‐targeting, facilitated by large hepatic blood flow, optimised organ microarchitecture to maximise surface exchange between blood and hepatocytes, and fenestrated sinusoid endothelium [164]. In addition, LNPs show natural liver tropism through adsorption of apolipoprotein E (ApoE) at the LNP surface in the bloodstream, mediating an uptake via low‐density lipoprotein (LDL) receptors on hepatocytes [165].

Key proof of concept studies implementing mRNA gene replacement strategy in vivo were first generated in animal models of methylmalonic acidaemia (MMA) (OMIM# 251000) [158, 163] and acute intermittent porphyria (AIP) (OMIM# 176000) [162]. MMA is a devastating systemic IMD caused by methylmalonyl‐CoA mutase (MUT) deficiency, a vitamin B12 dependent mitochondrial enzyme [166]. AIP is an autosomal dominant IMD caused by hepatic deficiency of a haeme biosynthesis enzyme, porphobilinogen deaminase (PBGD) [166, 167]. In both conditions, a single intravenous 0.5 mg/kg dose demonstrated efficacy. In two MMA mouse models (Mut−/−Tg^INS‐MCK‐Mu^ and Mut−/−Tg^INS‐CBA‐G715V^), robust hepatic protein expression was observed for at least 7 days following a single dose administration, confirmed by long‐term efficacy with repeated administrations in two different murine models, being observed for at least 12 weeks [158, 163]. The dose‐dependent hepatocytic expression of human PBGD following systemic administration of *h*PBGD‐encoding mRNA‐LNPs in the mouse model was followed by gradual normalisation of porphyrin precursors during both sporadic and recurrent neurovisceral attacks, as well as improvement in mitochondrial function, pain and motor impairment [162]. Repeated mRNA injections have shown better efficacy compared to current standard of care in a recently described non‐human primate (NHP) model of AIP [168].

The same dose of 0.5 mg/kg generated comparable 75%–80% hepatic expression of *hPBGD* in rats, rabbits and NHPs, with limited/no increase in the markers of liver toxicity, thus demonstrating translatability of mRNA‐LNP therapy in larger animals [162]. Therapeutic efficacy of mRNA was further demonstrated in a model of chemically induced porphyrin precursor accumulation in rabbits, which was used as a surrogate for clinical manifestation of variegate porphyria (VP) (OMIM 620483; https://www.omim.org/entry/620483?search=variegate%20porphyria&highlight=porphyria%2Cvariegate#176200) [169]. Sustained efficacy and tolerance with repeated administration in all species tested (mouse, rabbit and NHP) further showcased the translatability of the approach as repeat‐dose administration.

A preclinical study in a model of propionic acidaemia (PA) (OMIM# 606054) demonstrated the therapeutic potential of combinational mRNA therapy, using LNPs encapsulating an equimolar ratio of two different mRNAs, each encoding a different enzymatic subunit [157]. Propionic acidaemia (PA) is caused by a lack of propionyl carboxylase‐CoA carboxylase (PCC) function [166]. PCC is a heterododecamer enzyme consisting of α‐ and β‐subunits (PCCA and PCCB) [170, 171]. PCC deficiency can be caused by a deficiency in either subunit encoded by *PCCA* and *PCCB*, respectively, and due to their interdependency for stability [172]. In a hypomorphic PA murine model, LNP encapsulated, equimolar *PCCA* and *PCCB* mRNA encoding for both human PCCA and PCCB subunits was administered with repeat doses over 3 and 6 months, resulting in sustained efficacy and tolerability [157]. This was translated in a first‐in‐human phase I/II clinical trial with interim results showing promises of preclinical studies (detailed in the next section) [25].

Urea cycle disorders (UCDs), caused by inherited disorders of ureagenesis and nitrogen wasting, have also been targeted with mRNA‐LNP therapy and have shown great promise for citrin deficiency [173] (OMIM# 605814), argininosuccinic aciduria (ASA) (OMIM# 207900) [159, 160], ornithine transcarboxylase deficiency (OTCD) [174, 175] (OMIM# 311250) and arginase deficiency (OMIM# 207800) [161]. For example, mRNA therapy in a hypomorphic mouse model of ASA, the second most common UCD due to a lack of argininosuccinate lyase (ASL) enzyme activity, showed normalised survival and restored ureagenesis function after weekly systemic injections of *hASL* mRNA‐LNP from birth. This treatment also corrected additional secondary features of the disease including glutathione metabolism and glycogen storage. Additionally, rescue observed in mice treated as young adults further supported the therapeutic potential for late‐onset patients [160]. Neuroimaging in mRNA‐treated hypomorphic ASA mice suggested neurological benefits [176]. In arginase deficient mice, restored ureagenesis was associated with improvement of the cerebral pathology, including myelination, which prevented associated leukodystrophy and restored normal oligodendrocyte function [177].

Additional proof of concept studies have been published using mRNA‐LNP therapy in IMDs, with a range of short‐ and long‐term efficacy studies, such as α1‐antitrypsin deficiency (AATD) (OMIM# 613490) [178], classical galactosemia (OMIM# 230400) [179], very long chain acyl‐CoA deficiency (VLCAD) (OMIM# 201475) [180], medium chain acyl‐CoA deficiency (MCAD) (OMIM# 201450) [181], progressive familial intrahepatic cholestasis III (PFIC III) (OMIM# 602347) [182], tyrosinemia type I (OMIM# 276700) [183, 184], Fabry disease (OMIM# 301500) [185], maple syrup urine disease (MSUD) (OMIM# 248600) [186], glycogen storage disease type 1a (GSD1a) (OMIM# 232200) [187, 188] and phenylketonuria (PKU) (OMIM# 261600) [189]. All studies demonstrated varying extents of clinical correction ranging from restoration of functional proteins to therapeutically relevant levels alleviating or restoring the clinical phenotypes.

Safety assessments in these preclinical studies have continually demonstrated satisfactory tolerance to single and repeat dosing, supporting translation. These assessments include the reduction of liver toxicity markers such as alkaline phosphatase (ALP), aspartate aminotransferase (AST), alanine transaminase (ALT), blood urea nitrogen (BUN), and albumin in serum. In addition, cytokine panels such as interferon‐ƴ, TNF‐α, interleukin (IL)‐1β, and IL‐6 were measured, all demonstrating minimal to no inflammatory response [157, 158, 160, 161, 187, 189, 190].

Continual advancements in mRNA technology, such as sequence optimisation and chemical modifications, have enhanced translatability, minimised immunogenicity, and improved stability [141]. Recently a second‐generation mRNA drug targeting MMA (mRNA‐3705), where sequence elements in both the coding and untranslated regions of human methylmalonyl‐CoA mutase (hMMUT) were redesigned to improve protein expression, subcellular localisation, and minimise expression of antigen‐presenting cells (APCs), was tested. This resulted in significantly higher therapeutic efficacy, with 2.3‐fold higher liver MMUT protein expression detected [191]. These innovations demonstrate that mRNA‐based protein replacement therapy holds promise as a potential treatment option for rare genetic metabolic disorders that currently lack effective therapies. Further progress is already underway, with high‐throughput strategies now playing a key role in advancing optimisation of these therapies for clinical use. For instance, artificial intelligence‐based methods for screening of specialised ionisable lipids have shown promise for targeted delivery. This approach, which uses deep learning for lipid discovery and design, has significantly improved mRNA delivery potency to tissues such as muscle, lung, and nose [192]. Another novel development involves the use of translational pharmacokinetic/pharmacodynamic (PK/PD) models of mRNA therapeutics to predict starting doses for clinical studies targeting PA, MMA, and PKU [193, 194].

### 3.3 Clinical Trials

Following the rapid expansion of successful preclinical studies for liver‐based IMDs, first‐in‐man phase I/II clinical trials have been initiated. Seven trials were initially disclosed as being in set‐up, recruiting or completed for diverse indications. As of September 2025, four remain active, that is, MMA (NCT04899310), PA (NCT04159103), OTCD (NCT06488313), and GSD1A (NCT05095727) [195] (Table 1). Two have been terminated/withdrawn, and one currently has an unknown status (Table 1).

Interim results have been recently published for a PA trial (NCT04159103) [25]. Sixteen patients, aged 1.3–26.8 years, presenting with early onset of the disease, were enrolled and collectively received 340 intravenous doses. The main endpoint assessing safety was met with no dose‐limiting toxicity and only one Grade 3 severe adverse event, acute pancreatitis attributed to the underlying genetic disease, was reported. Five out of 16 (31%) patients developed infusion related reactions (IRRs) during the initial doses despite premedication with antipyretics and histamine receptor blockers. This was managed by slowing the infusion rate and adding corticosteroids. The IRRs resulted in the withdrawal of one patient, although this adverse event was not correlated to the dose administered.

Efficacy data showed a 70% reduction in the relative risk of presenting with a metabolic decompensation event (i.e., emergency care or hospitalisation). Although this was not significant (*p* = 0.09), these interim results are encouraging, particularly considering that they principally reflect cohorts receiving low doses and less frequent injections.

## 4 Other RNA‐Based Therapies

Although not detailed in this review, several exciting RNA‐based therapeutic advances have the potential to further revolutionise the way we treat IMDs. These include therapies targeting long non‐coding RNAs (lncRNAs), microRNA (miRNA), circular RNAs (circRNAs), small activating RNAs (saRNAs), and RNA editing technologies (Table 2). Targeting lncRNAs, which are distinct from mRNA in their biogenesis, localisations and functions, offers precise control over gene expression. Long non‐coding RNAs (lncRNAs) are gaining attention for their role in controlling metabolism through epigenetic and post‐transcriptional processes in IMDs. For example, in phenylketonuria (PKU), the lncRNAs HULC (in humans) and Pair (in mice) enhance PAH activity; Pair‐deficient mice developed PKU‐like traits [196, 197, 198].

**TABLE 2:** Overview of RNA‐based therapies for inherited metabolic disorders (IMDs).

Modality	Mechanism of action	Advantages	Challenges	Examples indicated
AONs	RNase H–mediated RNA cleavage: binds target RNA, recruit's RNase‐H to degrade RNA; Steric translation block: binds mRNA to block ribosomal access, inhibiting protein synthesis; Splice modulation: binds pre‐mRNA to redirect splicing, altering mRNA isoforms	High specificity; versatile; customisable and rapid design; can correct splicing defects; suitable for personalised n‐of‐1 approaches	Repeated dosing; delivery challenges; potential risk of toxicity	Mipomersen Inotersen
siRNAs	Integrates into RISC, guiding sequence‐specific mRNA cleavage to block protein synthesis	Potent, durable silencing; effective hepatocyte delivery with GalNAc conjugation; recycling of RISC sustains effect	Mainly limited to hepatocyte targeting; immune responses; endosomal escape barriers	Patisiran Inclisiran
mRNA	Engineered mRNA, delivered via LNPs, is translated in the cytoplasm for transient protein expression	Mutation agnostic direct protein restoration; avoids nuclear entry and genomic integration; scalable platform; strong proof of concept in IMDs	Transient expression requires repeated dosing; infusion reactions; immunogenicity risk; delivery beyond liver is challenging	mRNA‐3927
Other emerging RNA‐based approaches				
lncRNAs	Long noncoding RNAs (> 200 nt) regulate gene expression via chromatin remodelling, transcriptional modulation, mRNA stability, or antisense interactions	Versatile modes of regulation; can act as natural antisense transcripts (NATs), potential as therapeutic targets or biomarkers	Complicate therapeutic design, poorly conserved at primary sequence across species; delivery, tissue specificity, off‐target and immune issues	
miRNAs	Integrate into RISC to repress translation or destabilise mRNAs via partial complementarity	Broad regulatory potential, targeting multiple mRNAs. Mimics restore lost miRNA function; antagomirs block overactive miRNAs reversible; chemically stabilisable; biomarker utility	Delivery limits (esp. extrahepatic), off‐target effects, immune activation, chronic dosing. Complex roles in disease require precise target selection	
circRNAs	Closed RNA loops act as miRNA sponges, protein scaffolds, or translational regulators	High stability (exonuclease‐resistant); long half‐life; potential to modulate miRNA/protein networks in metabolic tissues; synthetic circRNAs engineered for stability	Complex biogenesis and specific roles complicate therapeutic design, limited understanding of translation efficiency, delivery challenges like other RNA therapies. Challenge in scalability Pro‐inflammatory	
saRNAs	Small dsRNAs target promoter regions, recruiting transcriptional machinery to upregulate gene expression (RNAa)	Specific activation of target genes, potential to upregulate deficient genes	Still early stage; mechanism less characterised; delivery challenges	
RNA editing	Site‐specific RNA base changes (A to I by ADARs, C to U by APOBEC, or programmable CRISPR‐based editors) to correct mutations or modulate expression	Precise, reversible correction without DNA change; avoids permanent genome edits; programmable systems enhance specificity	Off target risks due to non‐specific enzyme activity; delivery challenges and limited efficiency for some targets; early stage for IMDs. Immunogenicity against editor	

Regulating target gene expression at the post‐transcriptional level, miRNAs have emerged not only as an additional molecular mechanism in disease pathogenesis but also as biomarkers and therapeutic targets, as demonstrated in non‐alcoholic fatty liver [199]. miRNA studies in IMDs are currently limited and further research is still needed [200]. Similarly, circRNAs have gained attention for their unique ability to act as miRNA sponges, regulate transcription, modulate gene expression, and translocate proteins. Their tissue‐specific expression further underscores their therapeutic potential, particularly when targeted with AONs or siRNAs in metabolic disorders [201, 202].

In addition to down‐regulation, saRNAs provide a novel approach by activating the transcription of under‐expressed genes. By targeting promoter sequences, they allow for a targeted increase in enzymatic activity where it is deficient [203, 204, 205]. RNA editing or RNA modification technologies, such as antisense RNA‐guided adenosine deaminase acting on RNA (ADAR) based systems, enable the targeted correction of transcript‐level pathogenic mutations by modifying specific RNA sequences. These technologies offer a reversible and precise approach to addressing enzymatic defects in IMDs [206, 207].

Clinical promise has been observed from an early phase clinical study of clustered regularly interspaced short palindromic repeats and associated Cas9 endonuclease (CRISPR‐Cas9) genome editing (NTLA‐2001). This trial involved administering an LNP encapsulating mRNA for the Cas9 protein and a single guide RNA targeting *TTR* in a small group of patients with hATTR amyloidosis (NCT04601051) [208]. In vivo gene editing by CRISPR‐Cas9 has also been developed for use in preclinical studies of IMDs, including haemophilia B, hereditary tyrosinemia type I, OTC deficiency, and some lysosomal storage disorders [209].

The use of mRNA to encode nucleases for gene editing has shown promise in preclinical models [210, 211, 212] and has gained significant attention following successful delivery of a patient‐specific, LNP‐delivered base editing therapy in a patient with carbamoyl phosphate synthetase 1 deficiency [213]. The patient received two doses of an adenine base editing therapy, resulting in early signs of therapeutic benefit without any serious adverse events. Continued follow‐up will be essential to establish the long‐term safety, durability, and therapeutic efficacy of this approach.

These cutting‐edge advances complement the limitations of existing RNA technologies, offering durable and potentially curative solutions for IMDs that were once difficult or impossible to manage.

## 5 Conclusion

Advances in RNA‐based therapies have been transforming the treatment of IMDs, unlocking new possibilities and providing more effective treatment options for these rare genetic disorders. The evolution of RNA‐based approaches, including AONs, siRNAs, and mRNA, offers major potential for this diverse group of complex and varied disorders, many of which rely primarily on symptom management due to the lack of disease‐modifying treatments. Before these advancements, gene and cell therapies were the primary focus for the treatment of IMDs. However, RNA therapies offer a distinct advantage by not having to rely on the limitations associated with viral vectors. While RNA therapies have primarily targeted the liver and the central nervous system to date, ongoing research is expanding delivery to other organs.

RNA therapies may serve as valuable bridging strategies to more permanent interventions such as viral gene therapy or genome editing. Their inherent reversibility and dose adjustability enable early assessment of efficacy, safety, and therapeutic threshold, that is, the level of protein activity needed for clinical benefit thereby informing optimal dosing for more permanent therapies, de‐risking irreversible interventions while providing earlier access to treatment. The transient nature of RNA therapies also reduces the risk of permanent adverse effects, making them attractive where re‐dosing challenges limit viral vector–based approaches. For example, in AAV therapies where re‐administration is restricted, mRNA replacement therapy could support patients into adulthood, overcoming challenges such as the growing neonatal liver. Likewise, mRNA‐based strategies that transiently deliver gene‐editing enzymes may provide safer, reversible alternatives to permanent modifications, with temporary expression minimising long‐term risks.

Despite the transformative potential of RNA therapies, several challenges remain. The complexity of metabolic pathways means that altering the homeostasis of a protein can have widespread and often unpredictable effects, complicating therapeutic predictions. This challenge can be further exacerbated by variability and disease progression among individual IMDs. Additionally, while RNA therapies are now being successfully used in the clinic, translating preclinical proof‐of‐concept studies into successful clinical trials and ultimately approved drug products can be particularly challenging in rare diseases where financial constraints can impede progress. One exciting development is the growing acceptance of individualised n‐of‐1 or n‐of‐few targeted trials using RNA‐based approaches.

The flexibility of RNA technology enables the development of modular platforms, where validated chemistries, such as phosphorothioate, 2′‐MOE AONs, and GalNAc‐conjugated siRNAs or AONs can be consistently used while only the nucleotide sequence is altered. Delivery strategies can also be modularly adapted; for example, GalNAc conjugation for hepatocyte‐targeted IMDs. This modularity aligns with the efficiency of platform trials, which evaluate multiple interventions under a shared framework and common controls, improving efficiency, reducing regulatory burden, and accelerating translation [214]. A platform technology approach could allow rapid adaptation across diseases by leveraging shared processes in sequence selection, construct design, and delivery [215]. Integration of computational and systems‐based approaches with clinical data could further accelerate development. However, the modularity of RNA design may still raise safety challenges, as sequence‐related off‐target effects remain possible despite chemical modifications, necessitating careful design optimisation for chronic use [216].

Rapid regulatory approval plays a crucial role in enabling timely access to RNA therapies for IMDs, especially those life‐threatening conditions with aggressive disease progression. Accelerated mechanisms such as Breakthrough Therapy designation, adaptive trial designs and the FDA's Accelerating Rare disease Cures (ARC) program are streamlining rare disease development by integrating platform data, master protocols and cross‐referenced INDs [128, 217, 218]. Building on the IRDiRC n‐of‐1 roadmap, regulatory efficiency can be further enhanced through adaptive, platform‐based oversight frameworks that leverage shared preclinical data and cross‐referencing of master files across related INDs, while coordinated multi‐stakeholder dialogue can align evidence generation with early reimbursement planning [219]. Affordability may be improved through scalable manufacturing efficiencies, innovative outcome‐based pricing models, and early dialogue among developers, regulators, and payers to ensure sustainable and equitable access to patients [220].

RNA therapies may support the expansion of newborn screening (NBS) by enabling treatment for IMDs amenable to early intervention before irreversible pathology develops. The success of nusinersen in spinal muscular atrophy, where presymptomatic infants identified by genetic testing and increasingly through NBS achieve near‐normal motor development, provides a strong precedent [221]. Similarly, early initiation of lumasiran in infants with primary hyperoxaluria type 1 preserved renal function and improved nephrocalcinosis outcomes [222]. These examples demonstrate the feasibility and impact of RNA therapies when started early in life. Nonetheless, their transient nature requires life‐long dosing, with safety implications from long‐term repeated administration still uncertain and manufacturing costs potentially fluctuating over time. Despite these challenges, RNA therapies remain attractive for NBS‐linked implementation, as they can be rapidly deployed, adjusted, or discontinued before irreversible damage occurs. However, inclusion in screening panels will require careful, case‐by‐case evaluation based on disease severity, biomarker availability, and long‐term safety and efficacy [220].

## Author Contributions

All authors were involved in drafting and proofreading this article.

## Funding

The authors confirm independence from the sponsors; the content of the article has not been influenced by the sponsors. J.B. is supported by the Medical Research Council (Clinician Scientist Fellowship) (MR/T008024/1), MRC Transition Support Award (MR/Z504154/1), Great Ormond Street Hospital Charity (J.B., S.G., and R.G.), the Citrin Foundation and a research grant from Moderna Inc. (J.B. and S.G.). H.Z. is funded by the Medical Research Council (MR/Y008405/1), UKRI/Engineering and Physical Sciences Research Council (EP/Z536350/1), LifeArc, The Royal Society (IEC\NSFC\211238), SMA Europe, Great Ormond Street Hospital Charity (V4523), Rosetrees Trust (PGL24/100137), University College London Technology Fund (89‐315). P.B.M. is supported by University College London Technology Fund (89‐315), the Citrin Foundation, The Michael J Fox Foundation for Parkinson's Research (MJFF‐025709), and Great Ormond Street Hospital Charity (V2404). All authors are supported by the NIHR Great Ormond Street Hospital Biomedical Research Centre. The views expressed are those of the authors and not necessarily those of the NHS, the NIHR or the Department of Health.

## Ethics Statement

The authors have nothing to report.

## Consent

The authors have nothing to report.

## Conflicts of Interest

J.B. is in receipt of funding from The Medical Research Council, Great Ormond Street Hospital Children's Charity, Moderna Inc., and The Citrin Foundation and has received support from iECURE and the Citrin Foundation to attend meetings and/or travel. S.G. is in receipt of funding from Moderna Inc. and Great Ormond Street Hospital Children's Charity. There is no conflicts of interest from the other authors.

## Data Availability

The authors have nothing to report.
